# Flexible Capacitive Pressure Sensor Based on Microstructured Composite Dielectric Layer for Broad Linear Range Pressure Sensing Applications

**DOI:** 10.3390/mi13020223

**Published:** 2022-01-29

**Authors:** Yaoguang Shi, Xiaozhou Lü, Jihao Zhao, Wenran Wang, Xiangyu Meng, Pengfei Wang, Fan Li

**Affiliations:** 1 School of Aerospace Science and Technology, Xidian University, Xi’an 710071, China; shiyg@xidian.edu.cn (Y.S.); jihaozhao_xidian@outlook.com (J.Z.); 15029675366@163.com (W.W.); xymeng@stu.xidian.edu.cn (X.M.); 2 Qian Xuesen Laboratory of Space Technology, China Academy of Space Technology, Beijing 100094, China; wangpengfei@qxslab.cn; 3 Key Laboratory of Flight Techniques and Flight Safety, CAAC, Civil Aviation Flight University of China, Guanghan 618307, China; lifan@cafuc.edu.cn

**Keywords:** capacitive pressure sensor, microstructured dielectric composite layer, linearity, detection range

## Abstract

Flexible pressure sensors have attracted a considerable amount of attention in various fields including robotics and healthcare applications, among others. However, it remains significantly challenging to design and fabricate a flexible capacitive pressure sensor with a quite broad linearity detection range due to the nonlinear stress–strain relation of the hyperelastic polymer-based dielectric material. Along these lines, in this work, a novel flexible capacitive pressure sensor with microstructured composite dielectric layer (MCDL) is demonstrated. The MCDL was prepared by enforcing a solvent-free planetary mixing and replica molding method, while the performances of the flexible capacitive pressure sensor were characterized by performing various experimental tests. More specifically, the proposed capacitive pressure sensor with 4.0 wt % cone-type MCDL could perceive external pressure loads with a broad detection range of 0–1.3 MPa, which yielded a high sensitivity value of 3.97 × 10^−3^ kPa^−1^ in a relative wide linear range of 0–600 kPa. Moreover, the developed pressure sensor exhibited excellent repeatability during the application of 1000 consecutive cycles and a fast response time of 150 ms. Finally, the developed sensor was utilized for wearable monitoring and spatial pressure distribution sensing applications, which indicates the great perspectives of our approach for potential use in the robotics and healthcare fields.

## 1. Introduction

Flexible pressure sensors that are considered as the key component of the electronic skin with the unique ability to detect external pressure/force loads have attracted enhanced attention in recent years due to their widespread applications in health monitoring, wearable electronics, and robotics applications. It is interesting to notice that until now, different sensing mechanisms have been adopted in the literature to develop flexible pressure sensing components, such as piezoresistive [1,2,3], piezoelectric [4], capacitive [5,6,7], and triboelectric [8]. On top of that, during the past decade, numerous studies related to capacitive pressure sensors have been conducted due to their simple structure, facile signal acquisition, and low energy consumption [9]. By taking into account that the human skin has the ability to sense pressure over several orders of magnitude ranging from 10^1^ to 10^5^ Pa [10], we find that the ideal pressure sensor should be capable of sensing externally applied stress loads with high sensitivity in a wide linearity detection range. As a result, it is of great importance for both academic and industrial applications to devise such types of sensing elements with enhanced performance.

In addition, the sensitivity of the flexible capacitive pressure sensors could be promoted by introducing surface microstructures [11,12,13,14,15,16] inspired by the human skin or porous structures [17,18,19] within the dielectric layer. For instance, Niu et al. [11] proposed a flexible capacitive sensor configuration based on interlocked asymmetric nanocone arrays, which featured a high sensitivity value of 6.583 kPa^−1^ in a low-pressure region of 0–100 Pa. Xiong et al. [14] investigated a capacitive pressure sensor with micro-arrayed electrodes and an ultrathin dielectric layer, which demonstrated a detection range of 0–9 kPa, while the reported ultrahigh sensitivity of 30.2 kPa^−1^ could only be achieved in a narrow range of 0–130 Pa. Moreover, Qiu et al. [18] investigated a capacitive sensor with a three-dimensional (3D) graphene nanoplatelets/carbon nanotubes/silicone rubber/polyurethane sponge (GNPs/MWCNTs/SR/PS) dielectric layer, which exhibited a high sensitivity of 0.062 kPa^−1^ in the linear pressure range of 0–4.5 kPa. In consequence, the detection range of these highly sensitive capacitive pressure sensors with surface or embedded microstructures is still limited (<10 kPa) and should be further expanded. We have to underline that the employed dielectric composite, which possesses a relatively inherent superior dielectric constant than the conventional pure polymer dielectric materials, should be a promising choice in the development of the next generation of flexible capacitive pressure sensor with both high sensitivity and broad detection range [20,21,22,23,24,25,26,27,28]. For instance, Zhou et al. [22] developed a capacitive pressure sensor by utilizing a carbonyl iron particles/polydimethylsiloxane (CIP/PDMS)-based hair-like micro cilia array as a dielectric layer. Interestingly, the proposed sensor exhibited a high sensitivity of 0.28 kPa^−1^ in the range of 0–10 kPa, while the sensitivity was reduced to 0.02 kPa^−1^ in the range of 50–200 kPa. Liu et al. [26] proposed a capacitive pressure sensor based on sensitive element with hollow prism structure, and polydimethylsiloxane (PDMS), barium titanite (BaTiO_3_) and carbon nanotubes (CNTs) were used to synthesize the composite dielectrics. However, the sensor performed different physical behaviors at 0–20 kPa and 20–120 kPa pressure load, and the sensitivity of 0.1 kPa^−1^ could only be found at the pressure below 20 kPa. Therefore, in addition to the sensitivity and the total detection range, the linearity should also be improved for fabricating high-performance pressure sensors, which could lead into more accurate and reliable sensing elements, as well as easier signal processing. Furthermore, the limited linearity detection range usually originates as the result of the nonlinearity mechanical behavior of the polymer-based dielectric materials [13]. Thus, under the light of improving the sensitivity, linearity, and detection range simultaneously, the preparation of composite dielectric materials and the structural design with microstructures for the sensor should be promoted, which is the main objective of the present work.

Moreover, the fabrication method has significant impact on the characteristics of the composite, and the organic solvent that is usually utilized for dispersion of the nanofillers in composite dielectric materials preparation is ungreen [29]. Consequently, it is of great significance to come up with an environmentally friendly preparation method such as planetary mixing [2,19] or three-roll milling [7] for the polymer-based dielectric composite. In addition, carbon black (CB) and carbon nanotubes (CNTs) are ideal candidate materials to enhance the dielectric property of the PDMS due to their high aspect ratio and high dielectric constant [20,28]; thus, both these two nanofillers should be selected in preparing the dielectric composite to reduce the agglomeration of each single kind of nanofiller and increase the dispersion effect. 

Therefore, in this work, a novel flexible capacitive pressure sensor with microstructured composite dielectric layer (MCDL) is proposed. The different geometric structures of the flexible capacitive pressure sensor with MCDL were designed, and the sensing mechanism of the device was systematically analyzed. Meanwhile, the preparation of the dielectric composite material and the fabrication procedure of the capacitive pressure sensor were conducted. In addition, the responses of the flexible capacitive pressure sensor under pressures containing different microstructures and concentrations were characterized by performing experimental tests. Finally, the primary applications of the sensor for both wearable detector and spatial pressure distribution sensing were carried out.

## 2. Materials and Methods

### 2.1. Preparation of the CB/CNTs/PDMS Composite Prepolymer

**Figure 1:**
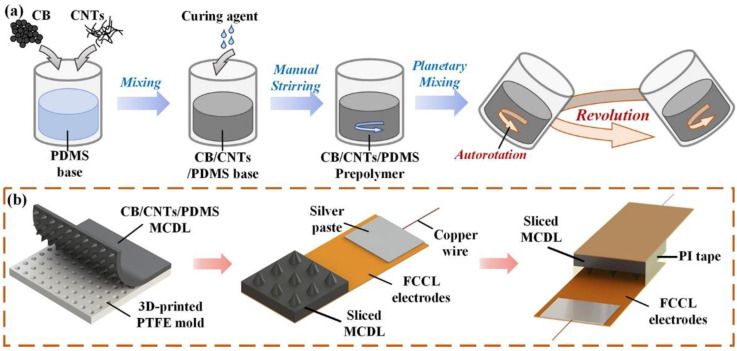
(**a**) Preparation process of the CB/CNTs/PDMS prepolymer through applying the planetary mixing method. (**b**) Fabrication procedure of the capacitive pressure sensor with MCDL.

Prior to the fabrication process of the flexible capacitive sensor, the carbon black/carbon nanotubes/polydimethylsiloxane (CB/CNTs/PDMS) composite prepolymer was prepared by enforcing a solvent-free mixing method. The CB and CNTs were chosen as the nanofiller elements of the dielectric composite. The conductive CB (Corpren-CB-3100, SPC Chemical Company, Muttenz, Switzerland) had a particle size of 30 nm and cetyltrimethylammonium ammonium bromide (CTAB) surface area of 1100 m^2^/g. The employed non-functionalized multiwalled CNTs (TNM3, Chengdu Organic Chemicals Co., Ltd., Chengdu, China) featured an outer diameter of 10–20 nm, length in the range of 10–30 μm, and specific surface area of >150 m^2^/g. As far as the compositions of the hybrid nanofillers are concerned, the filling weight ratio of CB and CNTs was determined as 1:1. The PDMS (Sylgard 184, Dow Silicones Corporation, Auburn, AL, USA) that was supplied as two-part liquid component kits was selected as the basis material. The preparation procedure of the CB/CNTs/PDMS composite prepolymer is schematically illustrated in Figure 1a. Firstly, a certain quantity of the hybrid fillers was weighed and mixed with the PDMS base polymer by manual stirring. Secondly, the curing agent was added into the mixture according to the mass ratio 1:10 of the PDMS base polymer and mixed manually. Thirdly, the mixture was mixed using the vacuum planetary mixer (HMV800, Shenzhen Hasai Technology Co., Ltd., Shenzhen, China) at room temperature for 5 min to simultaneously mix the mixture uniformly and remove the bubbles. The speeds of autorotation and revolution of the planetary mixer were set as 1600 rpm and 2000 rpm, respectively. Thus, the CB/CNTs/PDMS-based prepolymer obtained would be applied in following fabrication process of the flexible capacitive sensor.

### 2.2. Fabrication of the Flexible Capacitive Pressure Sensor

As is shown in Figure 1b, the detailed fabrication procedure of the flexible capacitive sensor with cone-type MCDL was carried out by enforcing the replica molding method. Firstly, the CB/CNTs/PDMS composite prepolymer was poured into a three-dimensional (3D) printed polytetrafluoroethylene (PTFE) mold with arrayed cone-type pits with a radius of 0.5 mm at the bottom, a depth of 1 mm, and a pitch of 1 mm. Secondly, the acquired composite prepolymer was degassed again and cured in a vacuum oven at 80 °C for 1.5 h. Subsequently, the cured MCDL was peeled off from the 3D printed mold, cleaned by ethyl alcohol, and dried. Thirdly, a conductive silver paste (CW2400, Chemtronics, Kennesaw, GA, USA) was screen-printed on the flexible copper clad laminate (FCCL) film with a width of 6 mm and thickness of 49 μm, which was utilized as flexible electrodes. Then the MCDL was cut into a square shape with a bottom area of 6 × 6 mm, while the copper wires were fixed to one edge of the FCCL electrodes by applying the brush painting technique and curing the silver paste at 80 °C for 20 min. Finally, the two FCCLs with copper wires were assembled with the MCDL by utilizing a 50 μm thick polyimide (PI) tape. The flat substrate surface of the MCDL was attached with the upper FCCL electrode by using the spray adhesive (Super 77, 3 M), and the other side of the dielectric layer with come-type microstructures was connected with bottom FCCL electrode by encapsulated with PI tape. The dimension of the PI tape was cut with same thickness of the MCDL, which ensures the microstructures keeping initial state without external loads applied. Moreover, the flexible capacitive sensors with both cylinder-type MCDL and planar composite dielectric layer (PCDL) were also manufactured with the same dimensions to compare their performances.

### 2.3. Characterization of the Dielectric Composite and the Pressure Sensor

The morphology of the CB/CNTs/PDMS-based composites was observed by using a ZEISS SIGMA Field Emission Scanning Electron Microscopes (FE-SEM). Additionally, the dielectric constant and the dielectric loss of the prepared CB/CNTs/PDMS-based composite materials were characterized from 100 Hz to 1 MHz by employing the high- and low-temperature dielectric temperature spectrum measurement system (DMS-2000, Partulab Technologies, Wuhan, China). As far as the mechanical characteristics of the CB/CNTs/PDMS-based MCDL and the cycling performance of the flexible capacitive pressure sensor are concerned, they were tested by the Tianyuan TY8000 series material test machine. First-order Ogden hyperelastic strain energy model was employed to fit the data for the purpose of studying the underlying deformation mechanism of the CB/CNTs/PDMS composite. On top of that, to investigate the deformation of the sensor under pressures, we used the commercial finite element analysis (FEA) software ABAQUS to model the PDMS-based composite dielectric layer with different microstructures under the application of various pressure loads. 

**Figure 2:**
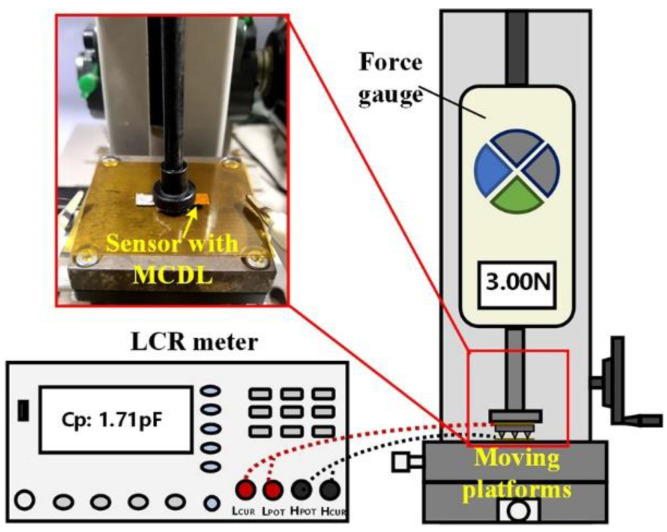
Experimental setup for performance characterization of the flexible capacitive sensor.

For carrying out the performance characterization of the developed flexible capacitive sensor, we employed a lab-made experimental setup, as shown in Figure 2. More specifically, the pressure-loading component consists of a digital high-precision force gauge (DS2-50N, ZHIQU), a manual test stand, and a moving platform. By moving the platforms, the force that is applied by the force gauge on the sensor varies, and the measuring range of the digital high-precision force gauge is 0–50 N with a high resolution of 0.01 N and accuracy of ±1%. The LCR meter (GFXY-5M, Guofeng) was used to measure the capacitive change under a constant frequency value of 1 MHz and DC voltage of 1 V.

## 3. Results and Discussion

### 3.1. Structure of the Flexible Capacitive Pressure Sensor

**Figure 3:**
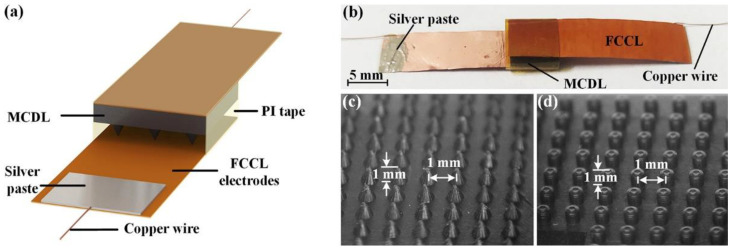
(**a**) Schematic diagram and (**b**) image of fabricated prototype of the capacitive sensor. Enlarged photographs of (**c**) cone-type MCDL and (**d**) cylinder-type MCDL.

A schematic diagram and image of the fabricated prototype of the capacitive pressure sensor with MCDL are revealed in Figure 3a,b, respectively. The flexible capacitive pressure sensor with parallel plate structural design consists of MCDL and flexible FCCL electrodes. The connected copper wires at the edges could significantly facilitate to connected with the instrument in the performance experimental test. Additionally, the cone-type and cylinder-type microstructure arrays of the MCDL manufactured by the replica molding method are displayed in Figure 3c,d, respectively. The MCDL made by replica molding method has a substrate of 6 × 6 × 1 mm with microstructures featuring a bottom radius of 0.5 mm, height of 1 mm, and pitch of 1 mm. It is interesting to notice that the identical feature of the microstructured array in MCDL could promote repeatable and consistent operation of the pressure sensor. Then, the total thickness of the fabricated prototype of the capacitive pressure sensor is 2.2 mm.

### 3.2. Dielectric Properties of the CB/CNTs/PDMS Composite

**Figure 4:**
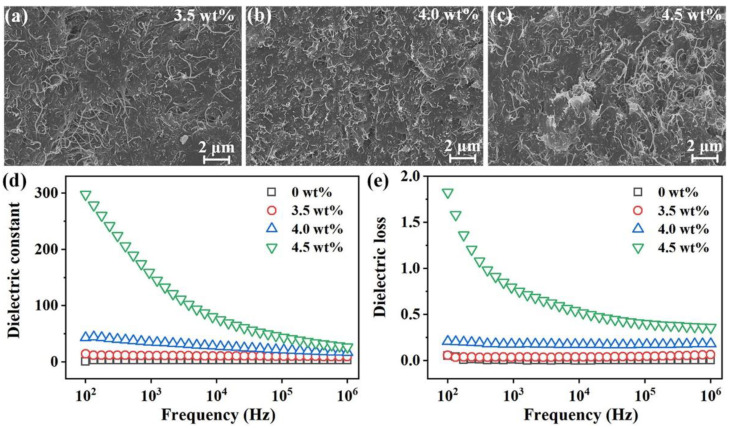
SEM images of the CB/CNTs/PDMS-based composite with different concentrations: (**a**) 3.5 wt %, (**b**) 4.0 wt %, and (**c**) 4.5 wt %. (**d**) Dielectric constant and (**e**) dielectric loss of the composites compared with pure PDMS.

The distribution of both the CB and CNTs within the PDMS matrix intrinsically affect the dielectric property of the composite, while the SEM image of the section of the 3.5 wt %, 4.0 wt %, and 4.5 wt % CB/CNTs/PDMS composites are shown in Figure 4a–c, respectively. From the acquired images, it can be seen that the CB and CNTs were well dispersed within the PDMS matrix for the composites with different nanofiller content. This result can be attributed to the relative high speed of the simultaneous autorotation and revolution procedures of the planetary mixing, which help to disperse the filler within the high-viscosity PDMS polymer matrix without organic solvent. 

Moreover, the dielectric properties of the composites hybridized with different contents of CB and CNTs (3.5 wt %, 4.0 wt %, and 4.5 wt %) were compared with that of the pure PDMS (0 wt %). As is disclosed in Figure 4d,e, the pure PDMS featured both the lowest dielectric constant and dielectric loss. With the increase of the contents of both CB and CNTs, the dielectric constant and dielectric loss of the composite became larger. It is interesting to notice that the application of an increasing frequency led to a reduction of the dielectric constant and dielectric loss characteristics. The improvement of dielectric constant with increasing the content of the conductive nanofillers was because of the number of micro capacitor networks formed by CB and CNTs particles closed to each other, which were separated by PDMS in the middle under electric fields [30]. For instance, the 4.5 wt % CB/CNTs/PDMS-based composite featured a higher dielectric constant of 297.53, while also exhibiting an excessive dielectric loss of 1.83 at the frequency value of 100 Hz. This increasement was caused by the enhanced interfacial polarization between the conductive nanofillers [30]. The corresponding dielectric constant and dielectric loss of the same composite were reduced to 26.71 and 0.36 at 1 MHz, respectively. Comparably, for the 3.5 wt % and 4.0 wt % CB/CNTs/PDMS-based composites, the dielectric constant was decreased to 9.52 and 16.65 at 1 MHz, respectively. However, the corresponding dielectric loss also diminished to 0.06 and 0.18, respectively. Thus, the 4.0 wt % composite was selected as the dielectric material for developing capacitive sensor because of its appropriate dielectric properties from 100 Hz to 1 MHz.

### 3.3. Sensing Mechanism of the Flexible Capacitive Pressure Sensor

**Figure 5:**
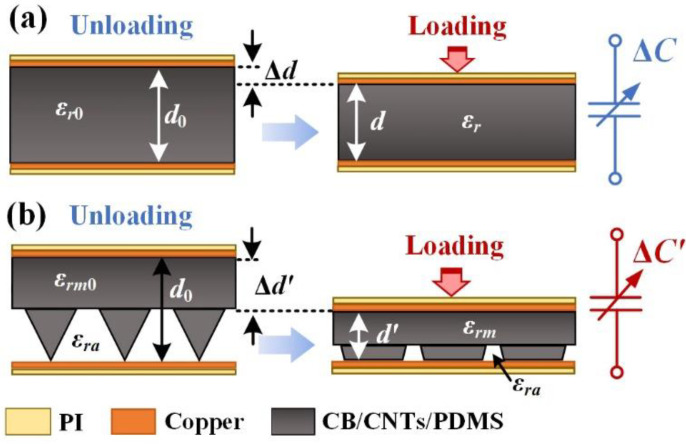
Depiction of the sensing mechanism of the flexible capacitive pressure sensor with (**a**) PCDL and (**b**) MCDL.

The underlying sensing mechanism of the capacitive pressure sensor with parallel plate structural design is shown in Figure 5. As can be ascertained from Figure 5a, since there are no microstructures on the substrate, the PCDL would be compressed when the external pressure is applied, which leads to the reduction of the distance between the parallel electrodes. Meanwhile, the dielectric constant that is related to the distance of the fillers in the PCDL should also be enhanced during the applied external loading. Therefore, the relative capacitive variation under applied pressure can be calculated as [31]

(1) $$\frac{\Delta C}{C_{0}}=\frac{{\;C-\;C}_{0}}{C_{0}}=\frac{\varepsilon_{0}\varepsilon_{r}\left(A/d\right)}{\;\varepsilon_{0}\varepsilon_{r0}\left({A/d}_{0}\right)}\;-\;1=\frac{\varepsilon_{r}}{\varepsilon_{r0}}\cdot \frac{d_{0}}{d}\;-\;1\propto r_{\varepsilon }\cdot r_{d}$$

where Δ*C* is the capacitance change of the pressure sensor with PCDL under the enforcement of a certain pressure; *C*_0_ and *C* are the capacitance values without and with the applied load on the device; and *d*_0_ and *d* represent the distance between the electrodes without and with applied pressure, respectively. Moreover, *ε*_0_ and *A* are the permittivity of the air (8.85 × 10^−12^ F/m) and the effective area of the two electrodes, respectively. *ε_r_* and *ε_r_*_0_ stand for the relative dielectric constant of the sensitive layer with and without loading, respectively, whereas *r_ε_* and *r_d_* are the variation ratio of the relative dielectric constant and distance of the capacitive pressure sensor under loading, respectively. Therefore, the relative capacitive variation of the sensor with PCDL presents a strong dependence on the variation in the dielectric constant and distance between the electrodes.

Similarly, the microstructures within the MCDL are uncompressed without pressure. When external pressure is applied, the microstructure array would be compressed, which leads to a reduction of the distance between the electrodes. Meanwhile, the dielectric constant that is related to the distance of the hybrid fillers in MCDL is anticipated to increase with the loading. The relative dielectric constant is combined with that of the composite and air in the gap between the electrodes, which are defined as *ε_rm_* and *ε_ra_* (≈1), as is illustrated in Figure 5b. Then, the relative dielectric constant of the sensor with MCDL $\varepsilon_{r}^{\prime }$ can be calculated by the following expression [25]:

(2) $$\varepsilon_{r}^{\prime }{=\varepsilon }_{rm}{\eta +\varepsilon }_{ra}\left(1\;-\;\eta \right)$$

where *η* is the volume ratio of the MCDL in the combined dielectric layer of the capacitive pressure sensor.

By substituting Equation (2) into Equation (1), one can calculate the relative capacitance change of the parallel plate capacitive pressure sensor with MCDL as below:

(3) $$\frac{\Delta C^{\prime }}{C_{0}^{\prime }}=\frac{\left[\varepsilon_{rm}{\eta +\varepsilon }_{ra}\left(1\;-\;\eta \right)\right]}{\left[\varepsilon_{rm0}\eta_{0}{+\varepsilon }_{ra}{(1\;-\;\eta }_{0})\right]}\cdot \frac{d_{0}}{d^{\prime }}\;-\;1\propto r_{\varepsilon }^{\prime }\cdot r_{d}^{\prime }$$

where ${\Delta C}^{\prime }$ is the capacitance change of the pressure sensor with MCDL under the enforcement of a certain pressure; $C_{0}^{\prime }$ is the initial capacitance without the application of any load on the device; $d^{\prime }$ represents the distance between the electrodes with loading; *ε_rm_*_0_ and *η*_0_ stand for the initial relative dielectric constant of the composite and initial volume ratio of the MCDL in the combined dielectric layer without loading, respectively; and $r_{\varepsilon }^{\prime }$ and $r_{d}^{\prime }$ signify the ratio of the relative dielectric constant and distance of the pressure sensor with MCDL after loading, respectively. Therefore, the performances of the capacitive sensor with MCDL are significantly affected by the variation of the relative dielectric constant of the composite, as well as the volume ratio. The corresponding Young’s modulus was 2.5 MPa, which is close to that of the pure PDMS (2.05 MPa) with mixing ratio of 10:1 [32]. Although the deformation of the pure PDMS would be a little larger, the variation of the dielectric constant should be more obvious for the CB/CNTs/PDMS dielectric composite. However, the influence of the variation of $r_{\varepsilon }^{\prime }$ as a function of the external load is hard to be analytically modeled. As a result, the performance of the sensor with MCDL should be further investigated by performing a thorough experimental characterization.

**Figure 6:**
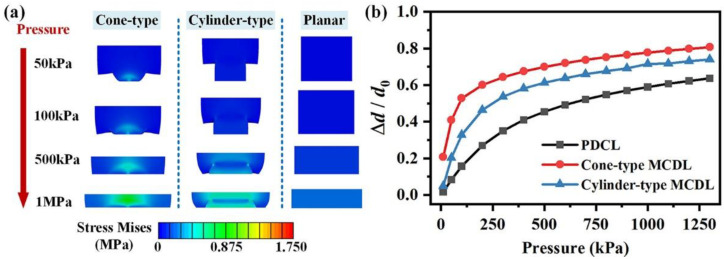
FEA analysis results of the deformation of the 4.0 wt % CB/CNTs/PDMS-based composite dielectric layer with different microstructures: (**a**) under the application of pressure ranging from 50 kPa to 1 MPa and (**b**) relative variation of the gap distance between the electrodes of the capacitive sensors under the enforcement of pressure from 0 to 1.3 MPa.

The volume ratio *η* of the MCDL would increase due to the compression of the air gap under loading. Thus, the *η* value should be larger than *η*_0_, which could be relevant to the distance between the electrodes. To investigate the mechanical performance of the MCDL under pressures, we conducted the uniaxial compression tests up to 25% strain for 4.0 wt % CB/CNTs/PDMS composite according to ISO 7743:2007 standard, and the test data were fit with different hyperelastic material models. The first-order Ogden model was found to fit the experimental data more accurately, and the corresponding parameters *μ* and *α* were 0.7859 and 4.0256, respectively. Figure 6a shows the FEA analysis results of the deformation of the 4.0 wt % CB/CNTs/PDMS-based composite dielectric layer with different microstructures under increasing pressure from 50 kPa to 1 MPa. Compared with the cylinder-type MCDL and PCDL structures, the compression that occurred in the cone-type microstructures was most significant. As the calculated results revealed in Figure 6b, the implementation of cone-type microstructures led to higher distance variation under a wide applied pressure range of 0–1.3 MPa. On the contrary, for the pressure sensor with PCDL or cylinder-type microstructures, the sensitivity of the sensor would be lower. Therefore, the flexible capacitive pressure sensor with cone-type MCDL exhibited greater $r_{d}^{\prime }$, which would promote the sensitivity performance.

### 3.4. Performance of the Flexible Capacitive Pressure Sensor

In the present work, the coefficient of determination (*R*^2^) was used to evaluate the linearity of the experimental results, while the sensitivity (*S*) could be given by the following expression [22]:

(4) $$S=\frac{{\delta (\Delta C/C}_{0})}{\delta P}$$

where *P* is the external pressure applied on the capacitive sensor. 

**Figure 7:**
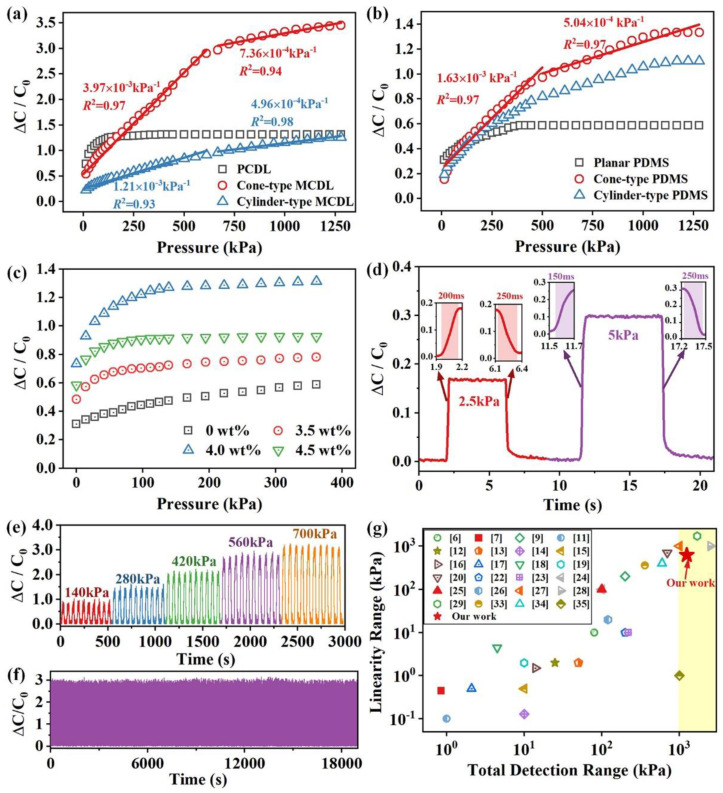
Relative capacitive variation as a function of different pressures of the sensors (**a**) with CB/CNTs/PDMS-based composite, (**b**) with PDMS, and (**c**) with PCDL as the dielectric layer. Response of the sensor with 4.0 wt % MCDL (**d**) under small pressures, (**e**) under the application of different pressures of 140−700 kPa for 10 cycles, and (**f**) under the enforcement of a pressure value of 600 kPa for 1000 cycles. (**g**) Linear detection range comparison with reported work [6,7,9,11,12,13,14,15,16,17,18,19,20,22,23,24,25,26,27,28,29,33,34,35].

As is divulged in Figure 7a, the sensitivity of the developed sensors with MCDL under different normal pressure was higher than that of the device with PCDL. More specifically, the flexible pressure sensor with 4.0 wt % cone-type MCDL possessed a sensitivity value of 3.97 × 10^−3^ kPa^−1^ in the range of 0–600 kPa, while it was reduced to 7.36 × 10^−4^ kPa^−1^ in the range of 0.6–1.3 MPa. Comparably, as far as the sensor with 4.0 wt % cylinder-type MCDL is concerned, the sensitivities were both reduced to the values of 1.21 × 10^−3^ kPa^−1^ in the range of 0–600 kPa and 4.96 × 10^−4^ kPa^−1^ in the range of 0.6–1.3 MPa, respectively. Furthermore, the flexible capacitive sensor with PCDL had the lowest sensitive detection range of 0–200 kPa. These results indicate that the cone-type microstructures on the MCDL play a key role in the sensitivity improvement compared with the PCDL, which is consistent with the above FEA analysis outcomes. On top of that, according to Equation (3), the manifestation of a large capacitance change of the sensor with MCDL could be also attributed to the enhancement of the dielectric constant of the dielectric composite, which is subjected to external pressure loads. However, the pressure sensor with cone-type MCDL exhibited a *R*^2^ value over 0.97, which was higher than that of 0.93 for the device with cylinder-type MCDL. The reason for this effect is the impact of the hyperelasticity in the cylinder-type microstructures should be more significant with larger volume ratio of polymer-based dielectric composite than that of the cone-type ones. 

In addition, Figure 7b compared the performance of the flexible capacitive sensor with the pure PDMS-based dielectric layer. It is interesting to notice that compared with the flexible pressure sensor with MCDL, the cone-type PDMS possesses both lower sensitivity of 1.63 × 10^−3^ kPa^−1^ and narrower linear detection range of 0–500 kPa than that with CB/CNTs/PDMS-based composite, while the sensitivity was further reduced to the value of 5.04 × 10^−4^ kPa^−1^ in the range of 0.5–1.3 MPa. Furthermore, the sensor with cylinder-type microstructure and planar PDMS still showed a lower sensitivity than that with cone-type microstructured PDMS in the same detection range. As a result, it can be concluded that the cone-type microstructures are beneficial in promoting sensitivity.

The relative capacitive variation of the sensors with PCDL under different pressures was also characterized to investigate the influence of the concentration of hybrid fillers on the performance, while the acquired results are displayed in Figure 7c. Therefore, the composite with 4.0 wt % hybrid fillers introduced higher sensitivity, which demonstrated that this concentration is suitable for capacitive sensing applications. However, all the fabricated sensors with PCDL and different concentrations had a poorer linearity range, which presented negligible variation when the pressure was beyond the value 200 kPa due to the recorded saturation of *r_ε_* of the composite material. According to Equation (1) and the FEA analysis in Section 3.3, the nonlinear relationship with the applied pressure of the sensor with PCDL could be attributed to the hyperelastic behavior in the PDMS-based composite. Thus, the flexible capacitive pressure sensor with 4.0 wt % cone-type MCDL should be chosen in the following characterization and applications.

In order to test the response characteristics of the proposed sensing elements, we loaded small normal stresses values of 2.5 kPa and 5 kPa on the sensor with 4.0 wt % cone-type MCDL for about 5 s and released the pressure applied, and the response time was recorded in the meantime. Figure 7d discloses the results of the sensor, while the smallest response time under loading and releasing of the pressure were 150 ms and 250 ms under 5 kPa, respectively. The extracted outcomes demonstrated that the cone-type microstructures were beneficial to the deformation and recovery of the pressure sensor. Moreover, the repeatability should be also investigated since it is very important for the practical application of the sensor. Figure 7e divulges the consecutive measurement results of the pressure sensor under a wide increasing range from 140 to 700 kPa for 10 cycles under each pressure. On top of that, the relative capacitive variation showed a highly reproducible and proportional manner, demonstrating that the sensor has excellent recovery capability. Figure 7f presents the output curve of the sensor under the pressure of 600 kPa during the application 1000 cycles (18 s per cycle). The experimental results indicated that the sensor has good repeatability, which could be ascribed to the good mechanical stability of the regular cone-type microstructure array. Moreover, the performance of the sensor with 4.0 wt % cone-type MCDL was compared with similar works that have been reported in previous literatures, as is shown in Figure 7g and Table 1. From the extracted comparative results, we can draw the conclusion that the developed sensor with cone-type MCDL in the present work featured competitive broader linear detection range.

### 3.5. Practical Application of the Flexible Capacitive Preeesure Sensor

**Figure 8:**
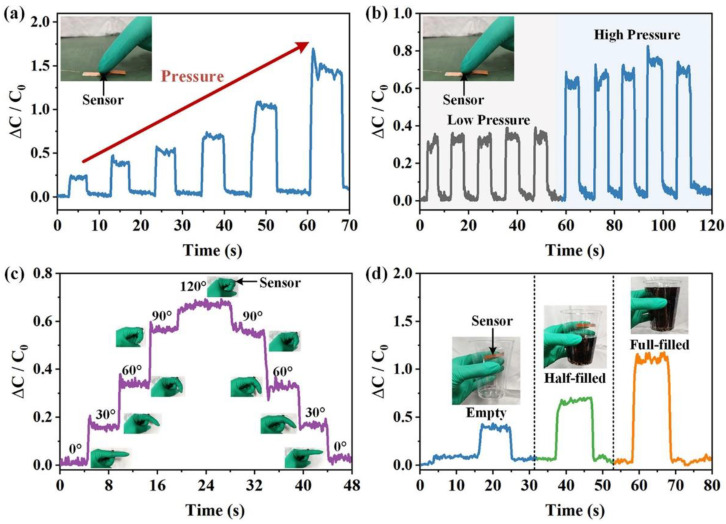
Applications of the capacitive pressure sensor for detection of (**a**) increasing pressures and (**b**) repeated pressures loaded by finger. (**c**) Response in capacitance of the sensor for finger bending angle motion detection. (**d**) Capacitance response of the sensor mounted on the gloves when grabbing and releasing the cup with different weights.

The practical applications of the developed flexible capacitive pressure sensor, as far as the wearable detection is concerned, were carried out. Under this perspective, the response of the capacitive sensor to different pressures applied by the fingertip is presented in Figure 8a, where it can be seen that as the pressure became enhanced, the relative capacitive increased monotonously. Moreover, the rapid response of the sensor indicated that the proposed device had the ability to return to the initial state after removing the fingertip. Furthermore, during the application of five cycles of repeated low or high pressure on the sensor by the fingertip, the developed sensor also featured good repeatability, as is shown in Figure 8b.

In addition to sensing the externally applied pressure force, the flexible capacitive pressure sensor has been also demonstrated in detecting the bending force. For that reason, we attached the developed pressure sensor on the joint of the index finger on the surface of a nitrile rubber glove so that it could be potentially used as a smart glove for detecting the bending state of the finger. As is illustrated in Figure 8c, by gradually bending the finger, the capacitance was progressively increased and can be employed to identify each bending state, as marked from 0 to 120 degrees. Similarly, the decrease of the capacitance value marked from 120 to 0 degrees represents the process of unfolding the finger. These results imply that the capacitive tactile sensor offers quite fast response/recovery times, as well as good stability in the bending states.

Moreover, to evaluate the detection capability under wearable tactile sensing applications, we finally attached the device to the fingertip for real-time weight monitoring, which was conducted by grabbing the plastic cup for 10 s and then releasing it. Figure 8d presents the dynamic response of the cup’s grabbing process empty, half-filled, and fully filled with drink. The response of the capacitive pressure sensor could recognize the state of the drink filled. More specifically, when the empty paper cup was grabbed, the relative change in the capacitance value was smallest as about 0.4. By increasing the amount of water, we found that the relative change in the capacitance of the sensor became significantly larger, reaching about 1.1 when the cup was fully filled with the drink. The linear response of the relative capacitive variation could be beneficial for the simplification of the detection circuit design.

**Figure 9:**
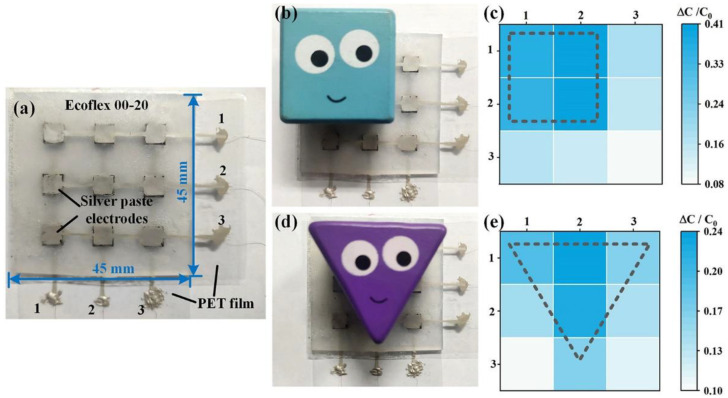
(**a**) Image of the 3 × 3 pixels capacitive pressure sensor array. Image and corresponding signal intensity and distribution of the sensor array with loading (**b**,**c**) cubic and (**d**,**e**) triangle blocks.

The ability to detect spatial pressure distribution might be critical to artificial electronic skin applications for intelligent robotics and prosthesis. Along these lines, a 3 × 3 pixelated CB/CNTs/PDMS-based capacitive pressure sensor array was designed and fabricated to detection of the spatial pressure distribution. The array had a dimension of 6 × 6 mm for each MCDL and 45 × 45 mm for the entire device, as is revealed in Figure 9a. The silver paste (CW2400, Chemtronics, Kennesaw, USA) was screen-printed on a polyethylene terephthalate (PET) substrate and played the role of the electrodes. The Ecoflex 00–20 rubber purchased from Smooth-On Inc. was cured and carved with nine square holes, which was utilized as the encapsulation of the MCDL array. Benefiting from the Ecoflex encapsulation and PET substrate, the deformation under external pressures was uniformly applied on the silver paste-based electrodes and MCDLs, which improves the reliability and cycle characteristics. Figure 9b,d shows the photograph of the placed cubic and triangle toy blocks on the pressure sensor array, respectively. The corresponding pressures applied were about 0.24 kPa and 0.22 kPa, respectively. Then, the pressure distribution on the sensor array was reflected by measuring the capacitive variations of each pixel. The color mapping of the relative capacitance variation in Figure 9c,e indicate that the placing position and force level of the blocks with different shapes can be well resolved by the sensor array in a sensitive manner.

## 4. Conclusions

In summary, in this work, a novel structural design of the flexible capacitive pressure sensor with MCDL was proposed. The CB/CNTs/PDMS-based composite polymer was prepared by applying the solvent-free planetary mixing approach, whereas the MCDL was fabricated by the replica molding method. Additionally, the performance of the capacitive pressure sensor with different microstructures was compared by FEA analysis and experimental characterization. The acquired results indicated that the cone-type MCDL plays a crucial role in improving the sensitivity of the sensor, and the device with 4.0 wt % possessed a wide range of 0–1.3 MPa, and a high sensitivity of 3.97 × 10^−3^ kPa^−1^ could be achieved in the comparably broad linear range of 0–600 kPa. Moreover, the response of the capacitive pressure sensor during the enforcement of 1000 loading-unloading cycles was repeatable and the response time could be as low as 150 ms. Finally, the primary wearable detection and spatial distribution sensing applications of the pressure sensor were carried out. Our work paves the way for the structural design and fabrication of flexible pressure sensors with enhanced sensitivity and broad linear detection range, which could be utilized for intelligent robotics and healthcare applications.

## Figures and Tables

**Table 1:** Performance comparison of the sensors in this paper and some other related works.

Ref.	Dielectric Materials	Sensitivity (kPa^−1^)	Pressure Range (kPa)	Linearity Range (kPa)
[7]	CNTs/PDMS	2.9	0.85	0.45
[11]	PVDF	6.583	1	0.1
[17]	BNF/PDMS	0.854	2.1	0.5
[18]	GNPs/MWCNTs/SR/PS	0.062	4.5	4.5
[14]	PVDF	30.2	10	0.13
[15]	AgNW/PDMS	0.831	10	0.5
[19]	CNTs/Ecoflex	6.42	10	2
[16]	PDMS	0.42	14	1.5
[12]	PVDF	0.35	25	2
[13]	CPDMS/PDMS	0.82	50	2
[6]	PDMS	0.06	80	10
[24]	PEDOT:PSS/PDMS	0.034	100	100
[25]	Conductive fabric/Ecoflex	0.0121	100	100
[26]	BaTiO_3_/CNTs/PDMS	0.1	120	20
[22]	CIP/PDMS	0.28	200	10
[9]	Air gap/ZnO NWs/PDMS	0.05645	200	200
[23]	PVA/H_3_PO_4_	3302.9	220	10
[33]	rGO/PVDF	0.02	360	360
[34]	PDMS	0.00001	600	400
[20]	CB/SR	0.00025	700	700
[35]	PDMS	1.15	1000	1
[27]	CIP/NdFeB/PDMS + CNT/PDMS	0.183	1000	1000
[29]	CNT/PDMS	0.065	1700	1700
[28]	CNTs/PDMS	0.0161	2550	1000
This Work	CB/CNT/PDMS	0.00397	1300	600
